# Risk factors for pancreatic cancer: underlying mechanisms and potential targets

**DOI:** 10.3389/fphys.2014.00490

**Published:** 2014-12-10

**Authors:** Mouad Edderkaoui, Guido Eibl

**Affiliations:** Medicine, Cedars-Sinai Medical CenterLos Angeles, CA, USA

**Keywords:** pancreatic cancer, risk factors, genetic mutations, inflammation, Src kinase

Pancreatic ductal adenocarcinoma (PDAC) is the fourth leading cause of cancer-related deaths among both men and women in the United States and remains essentially without effective therapies. The 5-year survival of patients with pancreatic cancer is a dismal 6% or less. Environmental risk factors such as smoking, diabetes, obesity, and alcoholism play major roles in the promotion of PDAC. However, currently we have limited understanding of how these risk factors promote the disease. The papers presented in this topic illustrate the latest knowledge regarding the mechanisms of pancreatic cancer induction and promotion by major risk factors such as smoking, pancreatitis, alcohol abuse, obesity and diabetes (Kolodecik et al., 2013), and less studied factors such blood group types (Pelzer et al., 2013) and the genetic mutations (Kong et al., 2013; Reznik et al., 2014; Weiss, 2014).

Indeed, an inherited predisposition to PDAC is believed to present in familial cancer syndromes such as the Peutz-Jeghers Syndrome, which is associated with germline mutations in the STK11/LKB1 gene, Familial Atypical Multiple Mole Melanoma syndrome, which results due to germline mutations in the p16/CDKN2A gene, Hereditary Breast-Ovarian Cancer syndrome (BRCA1/2 genes), Hereditary Non-polyposis Colorectal Cancer (mismatch repair genes), and Familial Adenomatous Polyposis syndrome (Reznik et al., 2014). Hereditary causes of pancreatitis, such as the autosomal dominant form caused by germline mutations of the cationic trypsinogen gene, PRSS1, have been indirectly linked to PDAC through early onset chronic pancreatitis with an associated 53-fold increased incidence and approximately 40% of hereditary pancreatitis patients noted to develop pancreatic cancer by age 70 (Weiss, 2014). Finally, more patients with blood group A suffer from PDAC whereas blood group O was less frequent in patients with PDAC (Pelzer et al., 2013). Kras mutations remain the most abundant genetic alteration found in pancreatic cancer patients. Kras mutations may lead to reducing power for ROS detoxification, leading to low ROS levels in pancreatic pre-neoplastic cells and in cancer cells. In adult stem cells and cancer stem cells, low ROS levels have been associated with the formation of a proliferation-permissive intracellular environment and with perseverance of self-renewal capacities. Therefore, it is conceivable that low intracellular ROS levels may contribute significantly to oncogenic Kras-mediated PDAC formation (Kong et al., 2013).

Specific gene-based, gene-product, and marker-based testing for the early detection of pancreatic cancer are currently being developed, with the potential for these to be useful as potential therapeutic targets as well.

Today, the role of stromal cells is highly appreciated for pancreatic cancer development. Immune cell infiltration into the tumor not only fail to contribute to disease eradication but rather due to exhibiting a Th2-type inflammation and immunosuppression is associated with more rapid disease progression, cachexia induction, and reduced survival. Polarization of macrophages toward M2-type correlates with a poor prognosis after surgery in resected patients. High CD163+ and CD204+ cell counts correlate with metastasis and poor prognosis in PDAC patients (Protti and De Monte, 2013; Tan et al., 2014).

Src kinase might serve as a critical mechanistic link between inflammation and cancer, mediating and propagating a cycle between immune and tissue cells that can ultimately lead to the development and progression of cancer (Liu et al., 2013). In addition, there is now compelling evidence that pancreatic stellate cells interact not only with cancer cells themselves, but with several other cell types in the stroma (endothelial cells, immune cells, and possibly neuronal cells) to promote cancer progression. Strategies to target the tumor microenvironment cells are proposed (Wilson et al., 2014). The central role of the mammalian target of rapamycin (mTOR) in mediating a crosstalk between the insulin/IGF-1 and GPCR signaling in pancreatic cancer cells is discussed in depth and strategies, including the use of metformin, to target this signaling pathway in PDAC cells are proposed (Rozengurt, 2014). Finally, a comprehensive review of the latest animal models of pancreatic cancer are discussed, proposing novel tools to study the mechanism of pancreatic cancer initiation and promotion by major risk factors.

## Conflict of interest statement

The authors declare that the research was conducted in the absence of any commercial or financial relationships that could be construed as a potential conflict of interest.
